# Proteolytic single hinge cleavage of pertuzumab impairs its Fc effector function and antitumor activity in vitro and in vivo

**DOI:** 10.1186/s13058-018-0972-4

**Published:** 2018-06-01

**Authors:** Hao-Ching Hsiao, Xuejun Fan, Robert E. Jordan, Ningyan Zhang, Zhiqiang An

**Affiliations:** Texas Therapeutics Institute, Brown Foundation Institute of Molecular Medicine, the University of Texas Health Science Center at Houston, 1825 Pressler St., Suite 532, Houston, TX 77030 USA

**Keywords:** Pertuzumab, HER2, Antibody hinge cleavage, Fc effector function, Breast cancer, Tumor invasion of humoral immunity

## Abstract

**Background:**

Proteolytic impairment of the Fc effector functions of therapeutic monoclonal antibodies (mAbs) can compromise their antitumor efficacy in the tumor microenvironment and may represent an unappreciated mechanism of host immune evasion. Pertuzumab is a human epidermal growth factor receptor 2 (HER2)-targeting antibody and has been widely used in the clinic in combination with trastuzumab for treatment of HER2-overexpressing breast cancer. Pertuzumab susceptibility to proteolytic hinge cleavage and its impact on the drug’s efficacy has not been previously studied.

**Methods:**

Pertuzumab was incubated with high and low HER2-expressing cancer cells and proteolytic cleavage in the lower hinge region was detected by western blotting. The single hinge cleaved pertuzumab (scIgG-P) was purified and evaluated for its ability to mediate antibody-dependent cellular cytotoxicity (ADCC) in vitro and anti-tumor efficacy in vivo. To assess the cleavage of trastuzumab (IgG-T) and pertuzumab (IgG-P) when simultaneously bound to the same cancer cell surface, F(ab’)_2_ fragments of IgG-T or IgG-P were combined with the intact IgG-P and IgG-T, respectively, to detect scIgG generation by western blotting.

**Results:**

Pertuzumab hinge cleavage occurred when the mAb was incubated with high HER2-expressing cancer cells. The hinge cleavage of pertuzumab caused a substantial loss of ADCC in vitro and reduced antitumor efficacy in vivo. The reduced ADCC function of scIgG-P was restored by an anti-hinge mAb specific for a cleavage site neoepitope. In addition, we constructed a protease-resistant version of the anti-hinge mAb that restored ADCC and the cell-killing functions of pertuzumab when cancer cells exressed a potent IgG hinge-cleaving protease. We also observed increased hinge cleavage of pertuzumab when combined with trastuzumab.

**Conclusion:**

The reduced Fc effector function of single hinge-cleaved pertuzumab can be restored by an anti-hinge mAb. The restoration effect indicated that immune function could be readily augmented when the damaged primary antibodies were bound to cancer cell surfaces. The anti-hinge mAb also restored Fc effector function to the mixture of proteolytically disabled trastuzumab and pertuzumab, suggesting a general therapeutic strategy to restore the immune effector function to protease-inactivated anticancer antibodies in the tumor microenvironment. The findings point to a novel tactic for developing breast cancer immunotherapy.

## Background

Previous studies have indicated that pathogen-associated and tumor-associated proteases are capable of cleaving human IgG1 within or adjacent to the hinge region [1–6]. For example, a group of tumor-associated proteases such as matrix metalloproteinase MMP3, MMP7, MMP9, and MMP12 generate limited cleavage of human IgG1 in vitro*,* and in some cases demonstrably in vivo. Such cleavage can confer substantial functional impairment to therapeutic antibodies [2, 4, 6]. In addition to F(ab’)_2_ fragments with their Fc domains removed, IgG1 antibodies with a single proteolytic cleavage in the lower hinge region (scIgG1), but with the Fc domain remaining attached, also exhibit impaired antibody-dependent cell-mediated cytotoxicity (ADCC) and complement-dependent cytotoxicity (CDC) [6–8]. We have demonstrated this susceptibility for trastuzumab in clinical tumor samples as shown with detection of single hinge-cleaved trastuzumab (scIgG-T) in tumor tissues from patients with breast cancer treated with trastuzumab as neoadjuvant [9].

In related investigations, it was shown that anti-hinge antibodies (AHAs) that specifically bind to the neoepitope formed by enzymatic scission successfully restored Fc-dependent function to cleaved therapeutic antibodies [7, 8, 10]. Polyclonal AHAs purified from human intravenous immunoglobulin (IVIG) was shown to restore function to a set of antigen-specific therapeutic monoclonal antibodies disabled by proteolytic hinge cleavage [8]. In a separate study, we were able to demonstrate strong ADCC restoration of scIgG-T by a monoclonal AHA [7]. In a model system using the potent IdeS protease (expressed by *S. pyogenes*), AHAs were also found to be subject to proteolytic attack in the hinge region with a resulting loss of restorative capability [7]. To address this issue, we applied a protein engineering approach to derive a protease-resistant monoclonal antibody (mAb). This version of an otherwise proteolysis-susceptible mAb retained the required Fc function in protease-rich environments [7, 11].

Pertuzumab (IgG-P) is a humanized mAb targeting human epidermal growth factor receptor 2 (HER2) [12] at an epitope different from that of trastuzumab (IgG-T) [13, 14]. Specifically, IgG-P interacts with domain II of HER2 whereas IgG-T targets domain IV of the HER2 receptor [13, 14]. It has been reported that ADCC is an important IgG-P mechanism of action [15–20]. There have been no reports of any previous study of IgG-P susceptibility to proteolytic hinge cleavage.

In this study, we demonstrated the occurrence of hinge cleavage of IgG-P in cell cultures and that the scission of a single peptide bond in this region diminished the anti-tumor activity and ADCC functions of IgG-P. We also found enhanced hinge cleavage for HER2-bound IgG-P when combined with trastuzumab. The latter observation pointed to a conceptual model to incorporate observations of basic biology, and suggests an application of that basic biological information to clinical situations in which polyclonal auto-antibodies are present against in situ tumor associated antigens (TAA). To this point, we investigated whether an AHA was effective at targeting both hinge cleaved IgG-T and IgG-P in combination to restore ADCC and antitumor activity. Taken together, our results suggest that using AHA to restore anticancer immunity is a promising strategy for developing a new class of breast cancer immunotherapy.

## Methods

### Cell culture and reagents

All cancer cell lines were obtained from the American Type Culture Collection (ATCC, Manassas, VA, USA) and maintained as previously described [7, 9]. Trastuzumab was obtained from a specialty pharmacy as previously described [7]. Pertuzumab with a single proteolytic cleavage in the lower hinge region (ScIgG-P) was prepared in house using a specific hinge cleavage proteinase (IdeS) (Sigma-Aldrich, St Louis, MO, USA). Intact IgG-P and protease-resistant IgG-P (PRIgG-P) were constructed based on variable sequences of pertuzumab, expressed in HEK293F cells, and purified using Protein A affinity chromatography as previously described [7]. Isotype control antibodies used in the study were prepared using the same expression system and protocols as the HER2 targeting IgG-P antibodies.

### Preparation of scIgG-P and F(ab’)_2_ fragments

Both scIgG-T and scIgG-P were prepared using IdeS partial cleavage by monitoring the disappearance of intact IgG using non-reducing SDS-PAGE detection. After the partial cleavage of IgG hinge, the mixtures of scIgGs and F(ab’)_2_ fragments were separated using Protein A agarose (ThermoFisher, Waltham, MA, USA) to elute the bound scIgGs and free Fc fragment from unbound F(ab’)_2_. Then CaptureSelect™ Kappa XL Affinity Matrix (ThermoFisher) was used to further purify F(ab’)_2_ fragments from the flow through of the protein A purification step, while in a separate step the free Fc fragment from Protein A elution was removed to enrich scIgGs from the CaptureSelect™ Kappa XL affinity Matrix. The purity of both scIgG and the F(ab’)_2_ was > 95% as shown on fast protein liquid chromatography (FPLC) size exclusion chromatography.

### IgG-P single-hinge cleavage when bound to a cancer cell line

SKBR3, BT474, MCF7-HER2, and MCF7 breast cancer cells and SKOV3 ovarian cancer cells were seeded on 6-well plates at 80% confluence and incubated for 24 h. The cancer cells were treated with 10 μg/ml of IgG-T, IgG-P, IgG-T F(ab’)_2_, or IgG-P F(ab’)_2_ for designated periods. Cells were harvested and lysed using radioimmunoprecipitation assay (RIPA) buffer (ThermoFisher) containing a 10% protease inhibitor cocktail (ThermoFisher). Monoclonal antibodies and F(ab’)_2_ fragments were enriched using Protein A (ThermoFisher) and their concentrations were determined as previously described [7]. Briefly, Protein A magnetic beads were incubated with cell lysates at 4 °C for 1 h, and the captured antibodies were collected in SDS containing sample buffer (Bio-Rad). Samples were subjected to SDS-PAGE and WB detection using a goat anti-human Fc-HRP conjugate (1:4000) (Jackson Immune Research Laboratory, West Grove, PA, USA) as previously described [7, 9].

### Detection of HER2 expression in breast cancer cell lines by flow cytometry

The cancer cells were detached using non-enzymatic solution (Fisher Scientific) from a cell culture flask and blocked in PBS buffer with 1% BSA for 45 min at room temperature. IgG-P was used to stain HER2 and R-PE (phycoerythrin) conjugated F(ab’)_2_ goat anti-human IgG Fcγ (1:200) (Jackson Immune Research Laboratory) was used as detection antibody. For the determination of the anti-hinge antibody binding to scIgG-P and scIgG-T on cancer cell surfaces, AHA (mAb 2095–2) was biotinylated and the binding of the AHA was detected using R-PE conjugated streptavidin (1:200) (Jackson Immune Research laboratories). All stained cells were analyzed by a Guava easyCyte HT flow cytometer according to the manufacturer’s instructions (Millipore, Hayward, CA, USA).

### Detection of CD4, CD8 and CD56 expression level in human peripheral blood mononuclear cells (PBMCs) cells by flow cytometry

CD4, CD8, and CD56 positive cells in PBMCs isolated from healthy human donors were detected by flow cytometry on a fluorescence-activated cell sorter FACScan (Becton Dickinson, Walpole, MA, USA). Alexa Fluor 700 anti-CD4 (eBioscience, San Diego, CA, USA), anti-CD8-Per-CP-Cy5.5, and anti-CD56-Per-CP-Cy5.5 (BD Pharmingen, San Diego, CA, USA) antibodies were used to detect expression levels of CD4, CD8, and CD56, respectively. Approximately 1 × 10^^6^ pelleted PBMC cells were blocked in PBS buffer with 1% BSA for 20 min at room temperature. The cells were then stained with antibodies at 4 °C for 30 min, washed twice in PBS buffer with 1% BSA and resuspended in 0.5 ml staining buffer for FACScan analysis.

### Mouse xenograft tumor model

All animal procedures and care were conducted in accordance with the animal care and use guidelines and the protocol was approved by the Animal Welfare Committee (AWC) of the University of Texas Medical School at Houston. Breast cancer cells (BT474) with high HER2 expression were prepared and implanted into athymic nude mice (*Foxn1*^*nu*^*/Fox1*^*+*^ genotype, Envigo, East Millstone, NJ, USA) subcutaneously (sc.) at the hind-leg fat pad to establish tumors as we described previously [7]. BT474 breast cancer cells (5 × 10^6^ cells/mouse) were implanted into 6 to 8 week old mice and antibody treatment was initiated after one additional week. The mAb treatments were performed once a week by intraperitoneal (ip) injection for 5 weeks at a dosage of 10 mg/kg body weight. Tumor growth and mouse health were monitored twice per week. Tumor growth was quantified by measuring the size of tumors using a Vernier scale caliper.

### Purification of human anti-hinge cleavage site antibodies from Octagam (IVIG)

A biotinylated human IgG1 hinge peptide analogue with the sequence biotin-THTCPPCPAPELLG (peptide 1981B) or a biotinylated IgG-P F(ab’)_2_ fragment (generated with the IdeS protease) were used as the absorbents to isolate human anti-hinge cleavage site autoantibodies from IVIG (pooled, purified IgGs from human plasma). The IVIG was diluted in PBS to a protein concentration of 1 mg/ml and was incubated with streptavidin agarose beads with bound peptide 1981B or biotinylated IgG-P F(ab’)_2_ for 1 h at 4 °C followed by three washes with PBS. Bound antibodies were eluted with 50 mM glycine (pH 2.6) then neutralized by adding 1/10th volume of 1 M Tris (pH 8.0). The antibody eluent was exchanged into PBS by adding 10× volume of PBS and concentrated using Amicon centrifugal filter units (MWCF, 30 kDa) (Millipore). Specificity enrichment of AHAP- F(ab’)_2_ was also performed by running the eluent through an additional affinity step with intact IgG-P linked on agarose. The flow through from the second enrichment step was buffer exchanged and concentrated using Amicon centrifugal filter units (MW, 30 kDa) (Millipore).

### Antibody-dependent cellular cytotoxicity (ADCC) assay

Polyclonal human AHAs and the monoclonal AHA (2095–2) were examined for their ability to restore ADCC activity using a non-invasive gold microelectrode-based cell cytotoxicity assay by the xCELLigence instrument (ACEA Biosciences, San Diego, CA, USA) as described previously [7]. SKOV3 and SKBR3 cancer cells were used as target cells (T) and human PBMCs, freshly isolated from two healthy donors, were used as effector cells (E) with the E:T ratio at 25:1. The degree of ADCC restoration by AHA coupled with scIgG-P was by comparison to the cells treated with IgG-P (30 nM), or scIgG-P (30 nM), respectively, with or without AHA (60 nM). The ADCC rescuing efficacy of polyclonal human AHAs or monoclonal AHA (2095–2 mAb) was measured by adding scIgG-P alone or in combination with scIgG-T together with a twofold to tenfold excess of AHAs. The percentage of cell lysis was defined as: (cell index of control group – cell index of treatment group)/cell index of control group) × 100. All experiments were replicated three times (*n* = 3).

### ELISA for assessing antibody binding to antigen HER2

A microtiter plate (ThermoFisher) was pre-coated with recombinantly expressed human HER2 extracellular domain protein (SinoBiological, Beijing, China) at 2 μg/ml overnight at 4 °C in PBS. Microtiter wells were washed with PBS and blocked with 200 μl/well of 3% BSA in PBS for 1 h at room temperature. Serial dilutions of IgG-P, PRIgG-P, or F(ab’)_2_ fragments were compared with the intact IgG-T/IgG-P antibodies for binding after incubating for 1 h at room temperature. After washing with PBS (three times), goat anti-human Fc-specific HRP conjugate (ThermoFisher) (1:4000) was used for detection with 3,3'-5,5' tetramethylbenzidin (TMB) (ThermoFisher) for 10 min incubation. The reaction was stopped by adding 50ul/well of 1 N H_2_SO_4_ and the individual wells were read for absorbance at 450 nm using a plate reader (SpectraMax M4, Molecular Devices, Sunnyvale, CA, USA).

### Statistical analysis

The pair-wise Student *t* test was used for statistical analysis using GraphPad software. Statistical significance was defined as a *p* value ≤0.05.

## Results

### Detection of IgG-P hinge cleavage when incubated with high HER2-expressing cancer cells

**Fig. 1:**
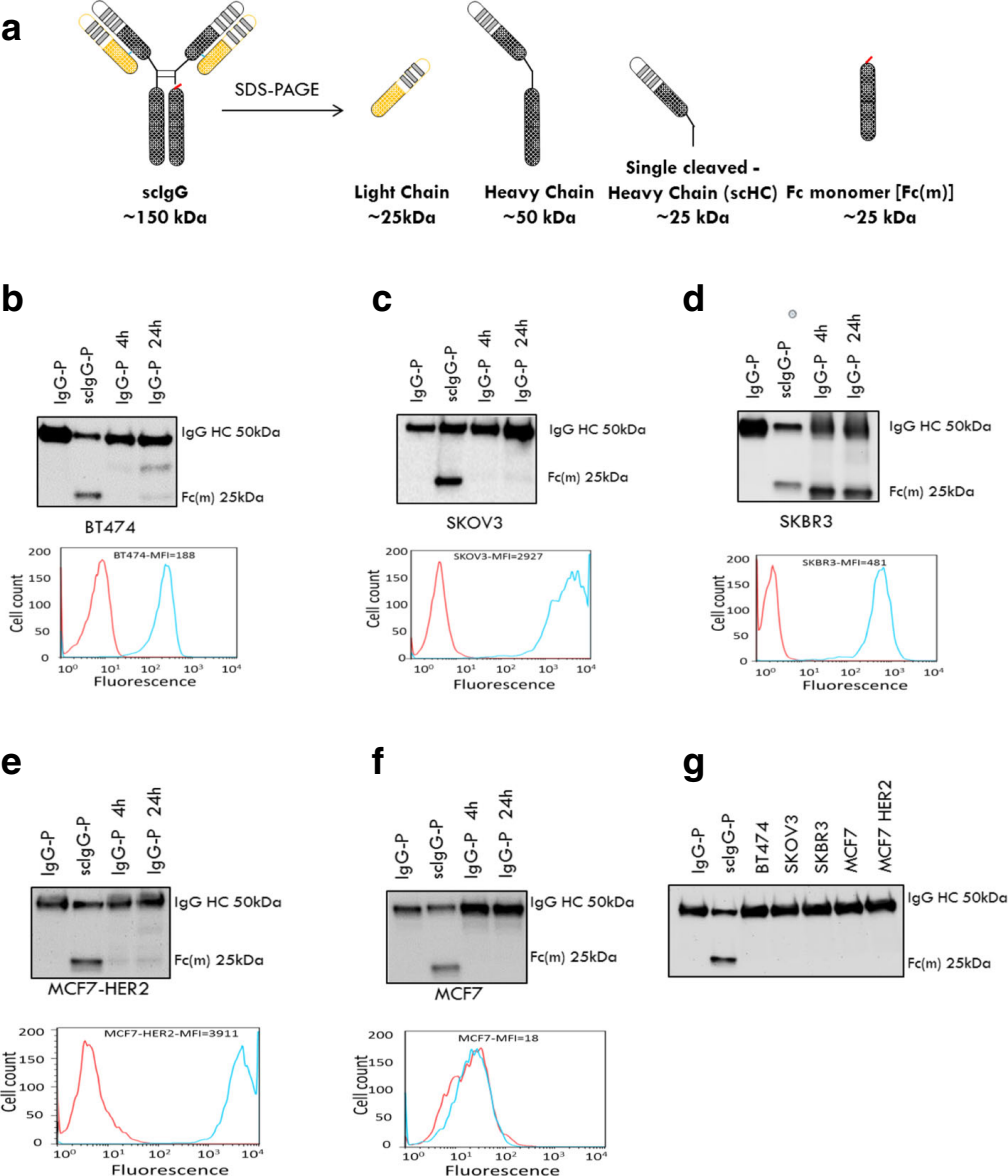
Pertuzumab (IgG-P) hinge cleavage was detected when IgG-P was incubated with higher human epidermal growth factor receptor 2 (HER2)-expressing cancer cell lines but not in low HER2 expressing cancer cell line. **a** Fragments of IgG with a single proteolytic cleavage in the lower hinge region (scIgG) generated under denaturing and reducing conditions, as assessed by western blotting detection. Fc(m) is the Fc monomer from the hinge cleavage and sc-Heavy chain indicates the N-terminal fragment from the hinge cleavage. Western blots showing hinge cleavage of IgG-P for the cell lines: BT474 (**b**, top panel); SKOV3 (**c**, top panel); SKBR3 (**d**, top panel); and MCF7-HER2, a MCF7 breast cancer cell line overexpressing HER2 (**e**, top panel). Low levels of Fc(m) were detected in MCF7 cells without HER2 expression (**f**, top panel). Cells were treated with 10 μg/ml of IgG-P for 4 h and 24 h at 37 °C, 5% CO_2_ in serum-free medium. Protein A magnetic beads were used to pull down the IgG-P proteolytic product. The hinge cleavage product, Fc monomer, was visualized by blotting the membrane using a secondary detection antibody, goat anti-human Fc-HRP antibody. A band shown on the western blotting with a molecular weight of 25 kDa was the Fc(m), which was seen in the scIgG-P enzymatically cleaved at the hinge region by immunoglobulin G-degrading enzyme S (IdeS). The intact IgG-P did not show a detectable band on the western blotting under reduced and denatured gel running conditions. High HER2 expression in BT474 (**b**, bottom panel), SKOV3 (**c**, bottom panel), SKBR3 (**d**, bottom panel), and MCF7-HER2 (**e**, bottom panel), and no detectable level of HER2 expression in MCF7 cells (**f**, bottom panel) were measured by FACS. **g** Detection of IgG-P hinge cleavage in cancer cell culture medium. Cancer cell-conditioned medium from BT474, SKOV3, SKBR3, MCF7, and MCF7-HER2 after treatment with IgG-P were collected after 24-h incubation and subjected to western blotting using a secondary detection antibody, anti-human Fc-HRP antibody: 10 μl of cancer cell-conditioned medium was loaded in each lane

As part of the ongoing investigation into whether antibody hinge cleavage represents a meaningful occurrence for IgG1 anticancer mAbs, we tested the hinge cleavage of pertuzumab (IgG-P) during incubation with high HER2-expressing cancer cells. As illustrated in Fig. 1a, the antibody with a single hinge cleavage (scIgG1) can be resolved into four components after separation by SDS-PAGE: light chain, full length heavy chain, hinge-cleaved heavy chain (scHC, upper fragment from the nicked hinge containing the Fab domain), and Fc monomer (Fc(m)). There was detectable Fc(m) in cell lysates after a 24-h incubation of IgG-P with high HER2-expressing cancer cells (BT474, SKOV3, SKBR3, and MCF7-HER2). IgG-P and scIgG-P were extracted from the cell lysates using Protein A beads and hinge cleavage, as indicated by presence of Fc(m), was tested by western blotting (WB) analysis using an anti-human Fc-specific detection antibody (Fig. 1b-e, top panels). SKBR3 cancer cells showed much stronger Fc(m) generation than the other high HER2-expressing cancer cell lines (Fig. 1d, top panel**)**. In contrast, low HER2-expressing MCF7 cancer cells and IgG-P incubated with conditioned medium from cell culture did not have detectable levels of Fc (m) (Fig. 1f, top panel and g). High HER2 expression in BT474, SKOV3, SKBR3, and MCF7-HER2 cells (Fig. 1b-e, bottom panels) were detected by FACS. In contrast, no HER2 expression was detected in MCF7 cancer cells (Fig. 1f, bottom panel). The latter result indicates that antibody hinge cleavage preferentially occurs on the cell surfaces when IgG-P engages its HER2 antigen target rather than in solution.

### Single hinge cleavage impeded the anti-tumor function of IgG-P

**Fig. 2:**
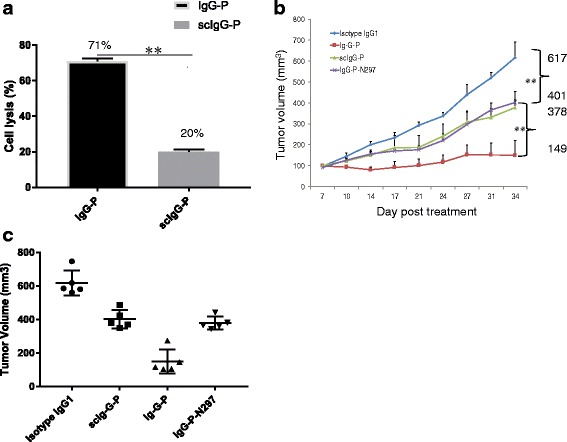
Single hinge cleavage caused a loss of antibody dependent cellular cytotoxicity (ADCC) activity in intact pertuzumab (IgG-P) that contributed to less tumor inhibition in IgG with a single proteolytic cleavage in the lower hinge region (scIgG-P) treatment group. **a** ADCC-targeted lysis of SKOV-3 ovarian cancer cells by IgG-P and scIgG-P was examined using the electrode impedance assay. SKOV-3 cells (5000 cells/well) were seeded on the E-plate as the target cell and peripheral blood mononuclear cells (25,000 cells/well) isolated from a single donor were used as the immune effector cells in complete cell culture medium containing scIgG-P (30 nM) and IgG-P (30 nM). The cell index after 96 h of incubation was the experimental end point (*n* = 3). The percentage of cell lysis was defined as: (cell index of control group – cell index of treatment group)/cell index of control group) × 100. **b** Tumor volumes from nude mice (*n* = 5) were inoculated subcutaneously with 5 × 10^6^ BT474 human breast cancer cells and treated with isotype IgG1 control, IgG-P, scIgG-P, or IgG-P N297A at 10 mg/kg weekly for a total of five doses until tumors reached an average size of 100mm^3^. **c** Tumor volumes at the end time point of the nude mice xenograft study for individual mice treated with isotype IgG1 control, IgG-P, scIgG-P, and IgG-P N297A. Tumor size was measured twice a week. The error bars in the graphs depict the standard deviation (SD) obtained in three independent experiments. *p < 0.05,**p < 0.01

It has been reported that ADCC is an important mechanism in the anticancer efficacy of pertuzumab [21]. To test whether proteolytic hinge cleavage of pertuzumab results in a loss of Fc-mediated cell killing function, we compared measurements of ADCC activity mediated by scIgG-P and intact IgG-P. We used a high HER2 expressing SKOV3 ovarian cancer cell line as the target and freshly isolated PBMCs as immune effector cells. The group treated with scIgG-P had significantly less lysis of cancer cells than the group treated with intact IgG-P (Fig. 2a). To compare scIgG-P antitumor function with the intact IgG-P in vivo, we adopted a murine xenograft tumor model in which mice were inoculated with an established high HER2-expressing cell line. Seven days after subcutaneous implantation of the cancer cells, tumor-bearing mice were randomly divided into groups (*n* = 5) for treatment with scIgG-P or IgG-P at a dose of 10 mg/kg, once weekly for five weeks. In addition to the isotype control IgG, IgG-P-N297A (IgG-P with a single amino acid mutation at position 297 to limit glycosylation of IgG-P) was used as a control group for a loss of Fc function. In comparison with the isotype control, all three pertuzumab antibody versions - scIgG-P, the N297A mutant, and intact IgG-P - inhibited tumor growth, but both scIgG-P and N297A mutant were significantly less effective than the intact IgG-P (Fig. 2b). With regard to the aglycosylated N297A mutant of IgG1, it has been established that this variant confers reduced Fc-mediated immune cell engagement and decreased ADCC due to impairment of Fc receptor binding [20]. Thus, the comparable reduction of tumor volume by scIgG-P and the aglycosylated IgG-P-N297A mutant pointed to a related mechanism of immune impairment (Fig. 2b). Tumor volumes at the end point of the xenograft study for individual mice in the four treatment groups are shown in Fig. 2c. The data further demonstrated that both the scIgG-P and the N297A mutant exhibited significantly less tumor inhibition efficacy than the intact IgG-P.

### Anti-hinge cleavage site autoantibodies (AHA) rescued the ADCC activity of scIgG-P

**Fig. 3:**
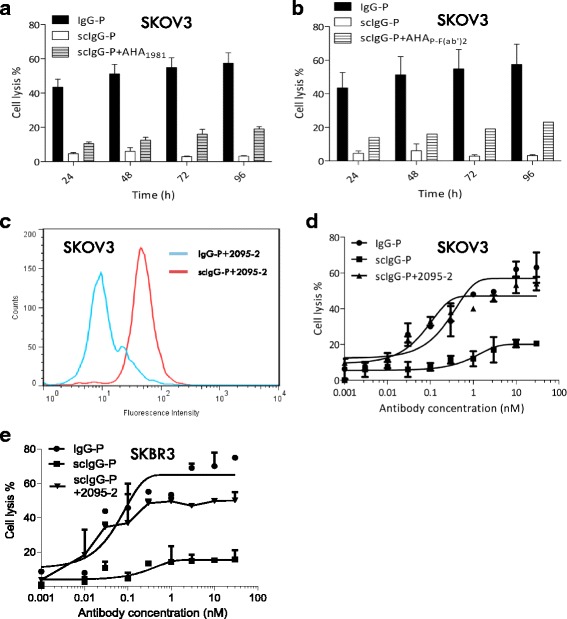
Anti-hinge antibodies rescued antibody dependent cellular cytotoxicity (ADCC) activity for single hinge cleaved pertuzumab (scIgG-P). **a-b** Purified human anti-protease-induced, anti-hinge autoantibodies (AHA) using peptide analogues representing hinge-immunoglobulin G-degrading enzyme S (IdeS) cleavage sites, 1981 or F(ab’)_2_ generated by digesting immunoglobulin G (IgG-P) with IdeS as the absorbent, restored ADCC activity for scIgG-P. SKOV-3 cell (5000 cells/well) was seeded on the E-plate as the target cell and peripheral blood mononuclear cells PBMCs (25,000 cells/well) isolated from a single donor were used as the immune effector cell in complete cell culture medium containing scIgG-P (30 nM). The percentage of cell lysis was defined as: (cell index of control group – cell index of treatment group)/cell index of control group) × 100. **c** Flow cytometry showing binding results for AH-mAb with IgG-P or scIgG-P on surfaces of high human epidermal growth factor receptor 2-expressing cancer cells. Biotinylated 2095–2 and streptavidin-PE conjugate were used for cell staining. **d-e** 2095–2 ADCC rescuing effect for scIgG-P at varying concentrations. A fixed concentration of 30 nM for IgG-P with threefold dilutions from 30 nM for 2095–2 were used in the ADCC assay. SKOV-3 cells (5000 cells/well) and SKBR3 cell (7000 cells/well) were used as the target cells and PBMCs isolated from a single donor were used as the immune effector cells at an effector (E)-target (T) ratio of 25:1

In a previous study, human AHAs were purified using F(ab’)_2_ affinity chromatography [8]. Those purified autoantibodies from IVIG restored biological functions to F(ab’)_2_ generated from a variety of monoclonal antibodies. In this study, we enriched AHA from IVIG using a peptide analogue of the point of IdeS cleavage of the human IgG1 hinge (peptide 1981 sequence ending in PAPELLG-_COOH_). AHA_1981_ demonstrated a degree of restoration of the ADCC activity of scIgG-P diminished by the IdeS protease (Fig. 3a). For comparison purposes, we also purified AHA from IVIG using IgG-P F(ab’)_2_ (generated with IdeS) as the absorbent and tested its ability to restore ADCC to F(ab’)_2_. As shown in Fig. 3b, AHA _P-F(ab’)2_ showed a comparable level of ADCC restoration to AHA_1981_. In previous studies, a monoclonal antibody AHA (2095–2) was shown to restore ADCC activity to scIgG-T [7, 10]. In this study, we investigated the analogous potential of using AH-mAb (2095–2) to rescue the function of scIgG-P. Indeed, the AH-mAb 2095–2 was strongly bound to scIgG-P on high HER2-expressing cancer cells (Fig. 3c), and as expected, restored the ADCC activity of scIgG-P to a level comparable with that of the intact IgG-P with SKOV3 ovarian cancer cells (Fig. 3d) or SKBR3 breast cancer cells (Fig. 3e) as the target cells.

### A variant of IgG-P, engineered to resist protease hinge cleavage, confirmed the impact of local protease action on IgG function

**Fig. 4:**
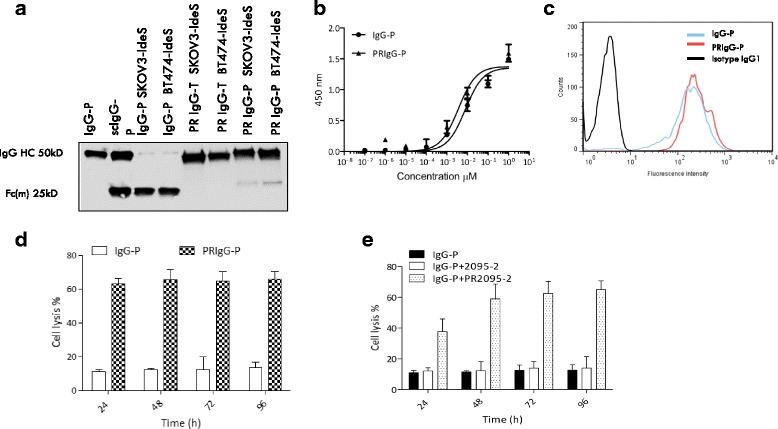
Pertuzumab variants with Fc engineered to withstand protease attack, protease-resistant variant of pertuzumab (PRIgG-P) and PR2095–2, restored lost antibody dependent cellular cytotoxicity (ADCC) activity for immunoglobulin G (IgG-P) in an immunoglobulin G-degrading enzyme S (IdeS)-rich environment. **a** The hinge cleavage profiles are shown for IgG-P, Protease-resistant variant of trastuzumab (PRIgG-T), and PRIgG-P for the SKOV3 ovarian cancer cell line overexpressing the IdeS protease (SKOV3-IdeS), and for the BT474 breast cancer cell line overexpressing IdeS protease-stable cell lines (BT474-IdeS). The SKOV3-IdeS and BT474-IdeS cancer cell lines were treated with 10 μg/ml of IgG-P/PRIgG-P/PRIgG-T for 24 h at 37 °C, 5% CO_2_ in serum-free medium. IgG-P and scIgG-P generated by digesting IgG-P with IdeS were used as standards. Protein A magnetic beads were used to pull down the IgG hinge proteolytic products, which were visualized by western blotting. **b** IgG-P and PRIgG-P binding affinity to human epidermal growth factor receptor 2 (HER2) receptor by ELISA. Microtiter plate wells were coated with recombinant human HER2 extracellular domain (ECD) at a concentration of 2 μg/ml as the antigen. IgG-P/PRIgG-P was used as the primary antibody then detected by goat anti-human Fc-HRP conjugate. **c** Flow cytometry showing the association between PRIgG-P or IgG-P and HER2 ECD on the cell surface. R-PE conjugated F(ab’)_2_ goat anti-human IgG Fcγ was used for detection. **d** Comparison of ADCC activity between IgG-P and PRIgG-P. SKOV-3-IdeS ovarian cancer cell line (5000 cells /well) was used as the target cell and peripheral blood mononuclear cells (PBMCs) (25,000 cells/well) isolated from a single donor were used as the immune effector cell. The percentage of cell lysis was defined as: (cell index of control group – cell index of treatment group)/cell index of control group) × 100. **e** Comparison of ADCC activity between 2095 and 2 and PR2095–2 in an IdeS-rich environment. The SKOV-3-IdeS cell (5000 cells/well) was used as the target cell and PBMCs (25,000 cells/well) isolated from a single donor were used as the immune effector cells. Fixed concentrations of 30 nM of IgG-P and 60 nM of 2095–2/PR2095–2, respectively, were used in the ADCC assay. Experiments were conducted in triplicate and the error bars in the graphs correspond to SDs obtained in three independent experiments

An engineered Fc variant of trastuzumab (PRIgG-T) was previously shown to withstand protease attack and to retain ADCC function in a protease-rich environment compared to IgG-T [7]. In this study, we constructed a protease-resistant variant of pertuzumab (PRIgG-P) using the same experimental approach. PRIgG-P demonstrated strong resistance to IdeS proteolysis compared to IgG-P when incubated with the protease-expressing BT474-IdeS and SKOV3-IdeS cells (Fig. 4a). As expected, PRIgG-P had similar binding to the antigen HER2 extracellular domain (ECD) as IgG-P (Fig. 4b, c). We investigated PRIgG-P antibody-mediated ADCC activity in cells with elevated proteolytic activity. The SKOV3-IdeS cell line was used as the target cell and PBMCs were used as the immune effector cell source. PRIgG-P clearly induced a higher percentage of cell lysis (> 60%) than IgG-P (< 20%) (Fig. 4d). Next, we examined the ADCC restorative function of a protease-resistant anti-hinge mAb, PR2095–2, in the IdeS-expressing cellular environment. Again, the SKOV3-IdeS cell line was used as the target cell and PBMCs were used as the source of immune effector cells. SKOV3-IdeS cells incubated with PR2095–2 and IgG-P had a higher percentage of cell lysis (~ 65%) than the group treated with 2095–2 and IgG-P (< 15%) at the end point of the experiment (96 h) (Fig. 4e). These results indicated a clear benefit of the engineered protease-resistant hinge for mAb-mediated ADCC in the IdeS protease-rich environment.

### Elevated IgG-P hinge cleavage occurred when IgG-T and IgG-P were combined

**Fig. 5:**
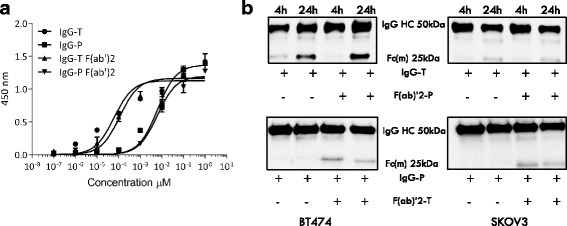
Intact trastuzumab (IgG-T) and intact pertuzumab (IgG-P) combination treatment increased IgG-P cleavage. **a**The binding affinity to human epidermal growth factor receptor 2 (HER2) extracellular domain (ECD) for IgG-P, IgG-T and F(ab’)_2_ fragments of IgG-T and IgG-P. Microtiter plate wells were coated with HER2 ECD at a concentration of 2 μg/ml as the antigen. Threefold dilutions of IgG-P, IgG-T and the F(ab’)_2_ fragments of IgG-T and IgG-P were each applied to microtiter wells coated with recombinant human HER2 ECD. Goat anti-human kappa light chain-HRP conjugate was used as the detection antibody. **b** IgG-T and IgG-P proteolytic cleavage profile with/without addition of IgG-P-F(ab’)_2_ fragment and IgG-T-F(ab’)_2_ fragment, respectively. BT474 breast cancer cell line or SKOV3 ovarian cancer cell line were treated with IgG-T (10 μg/ml) with/without F(ab’)_2_ fragment of IgG-P (10 μg/ml) or vice versa for 4 h and 24 h at 37 °C, 5% CO_2_ in serum-free medium. Protein A magnetic beads were used to pull down the IgG-P proteolytic product. The hinge cleavage product, Fc monomer, was visualized by blotting the membrane using a secondary detection antibody, goat anti-human Fc-HRP antibody

IgG-T and IgG-P are often used in combination in patients with breast cancer with high HER2 expression. To investigate how the hinge impairment of IgG-T and IgG-P affects the combination treatment, we assessed the cleavage of antibodies when simultaneously bound to the same cancer cell surface. For detection of scIgG generation, F(ab’)_2_ fragments of IgG-T or IgG-P were combined with the intact IgG-P and IgG-T, respectively. After incubation with either the BT474 or the SKOV3 cancer cell line, any detected Fc(m) must have derived from the intact IgG. This additive test system was made possible by the similarity in the binding affinity for HER2 ECD between the F(ab’)_2_ fragments and the corresponding full-length version of either IgG-P or IgG-T (Fig. 5a). Although it was not predicted in advance, the addition of the F(ab’)_2_ of IgG-T accelerated the generation of Fc(m) from IgG-P. This finding is unique in providing evidence for altered proteolytic kinetics of an antibody in a simultaneous binding circumstance. Intriguingly, there was not a corresponding increase in Fc(m) generation from IgG-T when combined with the F(ab’)_2_ of IgG-P (Fig. 5b). Structural rearrangements have been observed for IgG-T and IgG-P simultaneously interacting with HER2 ECD in an *in silico* analysis [22], which may explain the elevated IgG-P hinge cleavage in the presence of IgG-T.

### Anti-hinge cleavage site antibodies rescued ADCC activity with a mixture of scIgG-T and scIgG-P

**Fig. 6:**
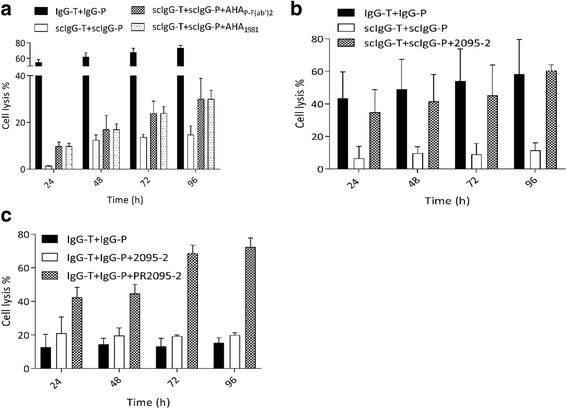
Anti-hinge cleavage site antibodies rescued antibody dependent cellular cytotoxicity (ADCC) activity for a mixture of single hinge cleaved trastuzumab (scIgG-T) and single hinge cleaved pertuzumab (scIgG-P). SKOV-3 cells (5000 cells/well) were seeded on the E-plate as the target cell and peripheral blood mononuclear cells (25,000 cells/well) isolated from a single donor were used as the immune effector cells in complete cell culture medium containing a mixture of intact pertuzumab (IgG-P) (30 nM) and intact trastuzumab (IgG-T) (30 nM), or scIgG-P (30 nM) and scIgG-T (30 nM) with and without anti-hinge antibody (AHA) (120 nM). The percentage of cell lysis was defined as: (cell index of control group – cell index of treatment group)/cell index of control group) × 100. **a** ADCC activity for a combination of IgG-T and IgG-P (black bar), a combination of scIgG-P and scIgG-T (white bar), and a combination of scIgG-T and scIgG-P using human anti-protease-induced AHA using peptide analogues representing hinge-immunoglobulin G-degrading enzyme S (IdeS) cleavage sites, 1981B (dark gray bar) or F(ab’)_2_ generated by digesting IgG-P with IdeS as the absorbent (light gray bar). **b** ADCC activity for a combination of IgG-T and IgG-P (black bar), a combination of scIgG-P and scIgG-T (white bar), and a combination of scIgG-T and scIgG-P using the anti-hinge mAb 2095–2 (dark gray bar). **c** ADCC cell lysis of the IdeS-expressing SKOV3-IdeS cell line by a combination of IgG-T and IgG-P (black bar), a combination of IgG-T and IgG-P + anti-hinge mAb 2095–2 (white bar), and a combination of IgG-T, IgG-P, and protease-resistant PR2095–2 (dark gray bar)

To determine whether the AHA can restore ADCC of scIgG-T and scIgG-P when used together on a HER2-expressing cell, we added purified human polyclonal anti-hinge autoantibodies (AHA_P- F(ab’)2_ or AHA_1981_) to a combination of scIgG-T and scIgG-P. As expected, the combination of scIgG-T, scIgG-P, and purified polyclonal human AHA produced a higher percentage of cell lysis than the target cell line (SKOV3) treated with the combination of scIgG-T and scIgG-P alone (Fig. 6a). Next, we examined the ADCC rescuing effect of AH-mAb (2095–2) for the scIgG-T and scIgG-P combination treatment. The target cell line (SKOV3) treated with scIgG-P and scIgG-T combined with 2095–2 showed a similar level of cell lysis as the group treated with intact IgG-P and IgG-T at all time points (Fig. 6b). This indicated that 2095–2 was able to access the single hinge cleavage site of the therapeutic antibodies in combination on the same cell surface and receptor. These results were extended to an examination of the restoration phenomenon in a protease-enriched setting. For this, the IdeS-expressing SKOV-IdeS cell line was used for the anti-HER2 combination and the parental and the protease-resistant versions of 2095–2 anti-hinge mAb were tested for ADCC restoration. In this case, PR2095–2, but not 2095–2, successfully rescued ADCC activity with the combination treatment (Fig. 6c). Thus, cell lytic functions of combined hinge-cleaved anti-HER2 mAbs were recovered by polyclonal and monoclonal anti-hinge antibodies in multiple settings.

## Discussion

The susceptibility of IgGs to functional inactivation by proteolytic enzymes has been studied in various ways including purified systems using cancer-associated enzymes, endogenous proteases expressed by tumor cells, and model cell lines with enhanced protease secretion. The present investigation touched on these aspects as they might relate to the considerable complexity of the in vivo tumor environment and therapeutic approaches used to treat it.

Pertuzumab (IgG-P) is often administered to patients with HER2-positive breast cancer together with trastuzumab (IgG-T) as combination therapy [12, 18, 20, 21]. Both IgG-P and IgG-T target HER2 but interact with different domains of HER2 [13, 14]. The present findings demonstrated that there was enhancement of IgG-P cleavage on the cell surface by endogenous proteolytic action when the mAb was used in combination with trastuzumab. In addition, the inherent sensitivity of IgG-P to the hinge cleavage was different from that for IgG-T. Substantial levels of hinge proteolysis of IgG-P were detected when IgG-P was incubated with SKBR3 cells, while IgG-T had lower sensitivity on this high HER2-expressing cancer cell line [9]. *In silico* data suggest a structural rearrangement of IgG-T and IgG-P when both mAbs are bound to the HER2 receptor simultaneously [22]. The present finding of interdependent protease susceptibility further extends the topological dynamics of the receptor. For example, the formation of the HER2-pertuzumab complex may cause rearrangement of the receptor-antibody complex to expose previously inaccessible proteolytic sites buried inside the antibody protein structure [5]. Structure-based methodologies likely will be needed to detail the interactions among the targeted antigen, therapeutic antibodies, and proteases.

Studies have implicated the involvement of Fc-mediated ADCC activity in IgG-P-mediated inhibition of tumor growth [15, 16, 20]. We earlier showed that the cleavage of a single peptide bond in the hinge caused a partial loss of the ADCC function of IgG-T in vitro and in vivo [7, 9]. In this study, we showed a similar reliance on Fc structural integrity for IgG-P-mediated ADCC effector function and tumor inhibition in vitro and in vivo. The single hinge cleaved IgG-P and an engineered immune cell engagement deficient mutant of pertuzumab (IgG-P N297A) showed decreased tumor inhibition. Our results suggest that IgG-P with a cleaved hinge partially impedes tumor inhibition due to the loss of Fc effector function. The partial inhibition of tumor growth by scIgG can be attributed to a lack of interference with the Fc-independent pathway of pertuzumab cell killing via HER2 antigen engagement.

We and others have reported that MMPs are associated with antibody hinge cleavage in tumor tissues [4, 9]. Numerous proteases coexist in a tumor microenvironment. This poses a hurdle for attributing IgG functional loss to particular enzymes or mixtures of enzymes [23]. Consequently, an alternative and well-defined model system was considered to be essential for the present study. The specificity and potency of IdeS for cleaving the IgG hinge enabled this attempt [6, 24–26]. This was confirmed by the demonstration that IgG-P was enzymatically cleaved at the hinge when incubated with IdeS expressing cancer cell lines and in the solution-phase. The precise peptide bond specificity of IdeS in targeting the hinge region of human IgGs led to the development or isolation of antibodies that specifically detect the presence of the hinge cleavage site. By extension of these findings, it is possible to consider therapeutic options for restoring IgG function by the association of a functional anti-hinge IgG to the site of IgG proteolysis in cell-bound IgGs. The concept is not limited to IdeS and can apply to physiologically relevant, cancer-related proteases in the tumor environment.

Anti-hinge autoantibodies can be found in healthy individuals and patients with inflammatory diseases [5, 27]. Indeed, purified autoantibodies prepared from serum IgGs using immobilized F(ab’)_2_ generated from IdeS-cleaved IgG-P as the absorbent or using immobilized peptide possessing the “…PAPELLG” sequence with the free C-terminal glycine showed modest restoration of ADCC activity to scIgG-P in vitro. These findings support the concept that endogenous anti-hinge autoantibodies, especially at enhanced levels, might be efficacious in certain disease circumstances. Further, the development of anti-hinge monoclonal antibodies to rescue compromised Fc-mediated functions in hinge-cleaved mAbs is a readily achievable approach for this purpose [6–8]. The monoclonal AHA 2095–2 used in this study targets the neoepitope of IdeS cleaved IgG [10] and can restore the ADCC activity of scIgG-T in vitro and also the inhibition of tumor growth by administering scIgGT in vivo [7, 10]. This study demonstrated that AHA 2095–2 restored ADCC activity of scIgG-P as well. Moreover, mAb 2095–2 restored function to both scIgG-T and scIgG-P when the two distinct, dysfunctional anti-HER2 mAbs were used in combination. Thus, these interconnected findings suggest substantial flexibility for AHA as a therapeutic approach for cancer treatment. In addition, a promising alternative strategy using an engineered protease-resistant hinge in trastuzumab was capable of overcoming the protease susceptibility of the original IgG. In protease-expressing cellular settings, PRIgG-T conferred resistance to proteolytic hinge cleavage both in vitro and in vivo [7]. In the present study, the concept was applied successfully to pertuzumab and to the anti-hinge mAb 2095–2 and suggests broad generality for this approach within the tumor environment.

## Conclusions

This study showed a readily detectable level of IgG-P hinge cleavage when incubated with high HER2-expressing breast cancer cell lines (but not with low HER2-expressing cells) and suggests that IgG proteolysis is facilitated when bound to the cell surface. ScIgG-P showed substantial loss of ADCC activity compared to un-cleaved IgG-P in vitro and was less potent against tumor growth in vivo. The loss of ADCC activity of scIgG-P can be restored by anti-hinge antibodies. An Fc engineering approach to derive a protease-resistant platform was shown to be applicable in two ways: (1) for directly maintaining IgG-P ADCC function in a protease-rich environment by engineering resistance into the heavy chain of IgG-P and (2) by the indirect method of engineering protease resistance into the AHA 2095–2. Both of these approaches afforded substantial protection in model systems to IgG-T and IgG-P singly or in combination. Taken together, the anti-hinge antibody and protease-resistant hinge suggest a powerful and versatile solution for overcoming the ability of tumor cells to evade the killing functions of targeted cancer immunotherapies.

## Abbreviations

ADCC: Antibody-dependent cellular cytotoxicity
AHA: Ant-hinge antibody
ATCC: American Type Culture Collection
AWC: Animal Welfare Committee
BSA: Bovine serum albumin
CDC: Complement-dependent cytotoxicity
ECD: Extracellular domain
ELISA: Enzyme-linked immunosorbent assay
Fab: Fragment antigen binding
FBS: Fetal bovine serum
Fc: Fragment crystallizable
Fc_(m)_: Fc monomer
HER2: Human epidermal growth factor receptor 2
HRP: Horseradish peroxidase
IdeS: Immunoglobulin G-degrading enzyme S
IgG: Immunoglobulin G
IgG-P: Intact pertuzumab
IgG-T: Intact trastuzumab
IVIG: Intravenous immunoglobulin
mAb: Monoclonal antibody
MMP: Matrix metalloproteinase
PBMC: Human peripheral blood mononuclear cells
PBS: Phosphate-buffered saline
PRIgG-P: Protease-resistant variant of pertuzumab
PRIgG-T: Protease-resistant variant of trastuzumab
RPMI 1640: Cell culture medium developed at Roswell Park Memorial Institute
scIgG-P: Single hinge cleaved pertuzumab
scIgG-T: Single hinge cleaved trastuzumab
SDS-PAGE: Sodium dodecyl sulfate polyacrylamide gel electrophoresis
TAA: Tumor-associated antigen
WB: Western blotting
